# Atlas of Material Damage. By George Wypych, ChemTec Publishing, Year 2012; 310 Pages. Price $325.00, ISBN 978-1-895198-48-5

**DOI:** 10.3390/ma6020565

**Published:** 2013-02-19

**Authors:** Shu-Kun Lin

**Affiliations:** MDPI AG, Kandererstrasse 25, CH-4057 Basel, Switzerland; E-Mail: lin@mdpi.com

The following paragraphs are reproduced from the website of the publisher [1].

*Atlas of Material Damage* has 464 microscopic pictures, schematic diagrams, and a few graphs, which show how materials fail, how they are produced to not fail, and how they are designed to perform particular functions to make outstanding products. Findings presented by each illustration are fully explained in the text and labeled.

In the recent past, products were distinguished by their formulations, which constituted highly guarded commercial secrets and know-how. Today, this is not enough. Materials, in order to compete, must have an optimal structure and specially designed morphology. This book gives numerous examples of how this special morphology can be achieved in electronics, the plastics industry, the pharmaceutical industry, aerospace, automotive applications, medicine, dentistry, and many other fields (see full list at the end).

It becomes clear from these examples that methods described by one branch of industry can be adapted by others. For instance, technology that powers the slow or targeted release of pharmaceutical products can be used successfully to prevent premature loss of vital additives from plastics.

Product reliability is the major aim of technological know-how. Uninterrupted performance of manufactured products at both typical and extreme conditions of their use is the major goal of product development and the most important indicator of material quality.

This book provides information on defects formation, material damage, and the structure of materials that must perform designed functions. The following aspects of material performance are discussed:

1. Effect of composition, morphological features, and structure of different materials on material performance, durability, and resilience
2. Analysis of causes of material damage and degradation
3. Effect of processing conditions on material damage
4. Effect of singular and combined action of different degradants on industrial products
5. Systematic analysis of existing knowledge regarding the modes of damage and morphology of damaged material
6. Technological steps necessary to obtain specifically designed morphology required for specific performance
7. Comparison of experiences generated in different sectors of industry regarding the most frequently encountered failures, reasons for these failures, and potential improvements preventing future damage.

This information is based on the most recent publications. Only 3% of the sources were published before 2000, and approximately65% appeared from2009–2012.

The name “Atlas” was selected to indicate the emphasis of the book on illustrations, with many real examples of damaged products and a discussion of the causes of damage, in addition to potential for material improvements.

This book should be owned and frequently consulted by engineers and researchers in the fields of: adhesives and sealants, aerospace, appliances, automotive, biotechnology, coil coating, composites, construction, dental materials, electronics industry, fibers, foams, food, laminates, lumber and wood products, medical, office equipment, optical materials, organics, metal industry, packaging (bottles and film), paints and coatings, pharmaceuticals, polymers, rubber and plastics, printing, pulp and paper, ship building and repair, stone, textile industry, windows and doors, wires and cables.

Professors and students interested in these subjects will require this book for a complete survey of modern technology.

Preface

In 1981, Carl Hanser Verlag published *An Atlas of Polymer Damage* by Lothar Engel, Hermann Klingele, Gottfried Ehrenstein, and Helmut Schaper. This unique publication quickly became my favorite, and was frequently consulted throughout the last thirty years.

In using it, I have learned that there are extensive applications of this knowledge, including:

• Materials can be made stronger and more durable with little or no cost by proper use of morphological structure
• In many cases, polymer additives can be eliminated
• A product’s service life can be extended
• Material damage can be avoided

These and other findings are discussed in this book, which is meant to be easy to read, as it is composed of hundreds of pictures and mechanisms of performance, with captions explaining what can be learned from the illustrations. The descriptions areas close to the observations of the original authors as permitted by the integrity of narration, since they have the privilege of viewing the information within a broader scope.

I expect that this book will generate a strong following because it opens so many unexploited possibilities to make what we use today even better. A number of recently introduced products adhere to these principles. Moreover, a great deal of research concentrates on using specially developed structural features for the betterment of properties of their materials. Many of today’s excellent products cannot be made without the application of the knowledge discussed in this book.

Users of the book will find that most of the research included was conducted between 2009 and today, thereby underlining the value of these findings, considering that many problems of the past are no longer relevant—they were not only solved, but the solutions have already been implemented in product manufacturing.

My goal was to produce a book that can add value to the previously published volume, since so much has changed in the last thirty years. Because it is clear from the analysis of a large number of research projects that structural knowledge and practical ideas are useful in very different ways, there is no limit to the applications made possible by this book.

## Table of Contents

1. Introduction

2. Material composition, structure, and morphological features

2.1. Materials having predominantly homogeneous structure and composition

2.2. Heterogeneous materials

2.2.1. Crystalline forms and amorphous regions

2.2.2. Materials containing insoluble additives

2.2.3. Materials containing immiscible phases

2.2.4. Composites

2.2.5. Multi-component layered materials

2.2.6. Foams, porosity

2.2.7. Compressed solids

2.3. Material surface versus bulk

3. Effect of processing on material structure

3.1. Temperature

3.2. Pressure

3.3. Time

3.4. Viscosity

3.5. Flow rate (shear rate)

3.6. Deformation

3.7. Orientation

4. Scale of damage. Basic concept

4.1. Atomic

4.2. Microscale

4.3. Macroscale

5. Microscopic mechanisms of damage caused by degradants

5.1. Bulk (mechanical forces)

5.1.1. Elastic-brittle fracture

5.1.2. Elastic-plastic deformation

5.1.3. Time-related damage

5.1.3.1. Fatigue

5.1.3.2. Creep

5.1.4. Impact damage

5.1.5. Shear fracture

5.1.6. Compression set

5.1.7. Bending forces

5.1.8. Anisotropic damage

5.2. Electric forces

5.2.1. Tracking

5.2.2. Arcing

5.2.3. Drying out in batteries

5.2.4. Pin-holes

5.2.5. Cracks

5.2.6. Delamination

5.3. Surface-initiated damage

5.3.1. Physical forces

5.3.1.1. Thermal treatment

5.3.1.1.1. Process heat

5.3.1.1.2. Conditions of performance

5.3.1.1.3. Infrared

5.3.1.1.4. Frictional heat

5.3.1.1.5. Low temperature effects

5.3.1.1.6. Thermal stresses

5.3.1.2. Radiation

5.3.1.2.1. Alpha and beta rays

5.3.1.2.2. Gamma rays

5.3.1.2.3. Laser beam

5.3.1.2.4. Cosmic rays

5.3.1.2.5. Plasma

5.3.1.3. Weathering

5.3.2. Mechanical action

5.3.2.1. Scratching

5.3.2.2. Impact

5.3.2.3. Adhesive failure, sliding, rolling

5.3.3. Chemical reactions

5.3.3.1. Molecular oxygen

5.3.3.2. Ozone

5.3.3.3. Atomic oxygen

5.3.3.4. Sulfur dioxide

5.3.3.5. Particulate matter

5.3.3.6. Other gaseous pollutants

5.4. Combination of degrading elements

5.4.1. Environmental stress cracking

5.4.2. Biodegradation and biodeterioration

5.4.3. Effect of body fluids

5.4.4. Controlled-release substances in pharmaceutical applications

5.4.5. Corrosion

* *Editor’s Note*: The brief summary and the contents of the books are reported as provided by the author or the publishers. Authors and publishers are encouraged to send review copies of their recent books of potential interest to readers of *Materials* to the Publisher (Dr. Shu-Kun Lin, Molecular Diversity Preservation International (MDPI), Kandererstrasse 25, CH-4057 Basel, Switzerland. Tel. +41 61 683 77 34; Fax: +41 61 302 89 18, E-mail: lin@mdpi.org). Some books will be offered to the scholarly community for the purpose of preparing full-length reviews.
